# WIN site inhibition disrupts a subset of WDR5 function

**DOI:** 10.1038/s41598-022-05947-9

**Published:** 2022-02-03

**Authors:** Andrew J. Siladi, Jing Wang, Andrea C. Florian, Lance R. Thomas, Joy H. Creighton, Brittany K. Matlock, David K. Flaherty, Shelly L. Lorey, Gregory C. Howard, Stephen W. Fesik, April M. Weissmiller, Qi Liu, William P. Tansey

**Affiliations:** 1 Department of Cell and Developmental Biology, Vanderbilt University School of Medicine, 465 21st Avenue South, Nashville, TN 37232 USA; 2 Department of Biostatistics, Vanderbilt University Medical Center, Nashville, TN 37232 USA; 3 Center for Quantitative Sciences, Vanderbilt University Medical Center, Nashville, TN 37232 USA; 4 Vanderbilt University Medical Center Flow Cytometry Shared Resource, Vanderbilt University Medical Center, Nashville, TN 37232 USA; 5 Department of Biochemistry, Vanderbilt University School of Medicine, Nashville, TN 37232 USA; 6 Department of Pharmacology, Vanderbilt University School of Medicine, Nashville, TN 37232 USA; 7 Department of Chemistry, Vanderbilt University, Nashville, TN 37232 USA; 8 Present Address: Oncocyte Corporation, 2 International Drive, Suite 510, Nashville, TN 37217 USA; 9 Present Address: Department of Biology, Middle Tennessee State University, Murfreesboro, TN 32132 USA

**Keywords:** Cancer therapy, Transcription

## Abstract

WDR5 nucleates the assembly of histone-modifying complexes and acts outside this context in a range of chromatin-centric processes. WDR5 is also a prominent target for pharmacological inhibition in cancer. Small-molecule degraders of WDR5 have been described, but most drug discovery efforts center on blocking the WIN site of WDR5, an arginine binding cavity that engages MLL/SET enzymes that deposit histone H3 lysine 4 methylation (H3K4me). Therapeutic application of WIN site inhibitors is complicated by the disparate functions of WDR5, but is generally guided by two assumptions—that WIN site inhibitors disable all functions of WDR5, and that changes in H3K4me drive the transcriptional response of cancer cells to WIN site blockade. Here, we test these assumptions by comparing the impact of WIN site inhibition versus WDR5 degradation on H3K4me and transcriptional processes. We show that WIN site inhibition disables only a specific subset of WDR5 activity, and that H3K4me changes induced by WDR5 depletion do not explain accompanying transcriptional responses. These data recast WIN site inhibitors as selective loss-of-function agents, contradict H3K4me as a relevant mechanism of action for WDR5 inhibitors, and indicate distinct clinical applications of WIN site inhibitors and WDR5 degraders.

## Introduction

WDR5 is a highly-conserved protein that performs a variety of functions in the nucleus^1^. Its best-known role is scaffolding the MLL/SET complexes that catalyze histone H3 lysine 4 di- and tri-methylation (H3K4Me2/Me3), but WDR5 acts outside this setting to promote ribosomal protein gene transcription^2^, recruit MYC to chromatin^3,4^, and bookmark genes for reactivation after mitosis^5^. WDR5 is also overexpressed in cancer, and is an auspicious target for pharmacological inhibition in malignancy^1^. Most drug discovery efforts focus on blocking the “WIN site” of WDR5^6–12^, an arginine-binding cavity that tethers WDR5 to chromatin and recognizes an arginine-containing “WIN motif” in partner proteins such as the MLL/SET family of histone methyltransferases^13^, KIF2A^14^, the kinase PDPK1^15^, and others^1^. Although in vivo studies are limited by the poor drug-like characteristics of extant WIN site inhibitors, in vitro profiling demonstrates that small molecule WIN site blockers can inhibit cancer cells carrying oncogenic mutations in MLL1, C/EBPα, p53, and MYC^2,6–10,12^, forecasting that WIN site inhibitors could have widespread utility as anti-cancer agents.

Successful application of WIN site inhibitors will require understanding the role of the WIN site in tumor cell processes and knowing precisely how WIN site blockade leads to a cancer cell response. This information is essential to apply WIN site inhibitors as a targeted therapy, to define patient selection criteria, and to predict therapeutic windows and on-target toxicities. In modern drug discovery, this understanding often comes from probing the impact of long-term loss or depletion of the target protein on the cancer cell milieu and from correlative studies that tie phenotypic responses to a canonical molecular function of that target^16^. And WDR5 is no exception. RNAi-mediated knockdown is often used to predict whether WIN site inhibitors will have activity in specific contexts, and changes in H3K4 methylation almost always assumed to drive the molecular mechanism of response^6,7,17–22^. But knockdown studies conflate blocking the WIN site of WDR5 with loss of the entire protein, and because WDR5 is pan-essential^23^ cannot explain how WIN site inhibitors show cancer cell-selective inhibition in vitro^6,11,17^. Moreover, linking WIN site inhibition to changes in H3K4me fails to discriminate between H3K4me as a mark of active chromatin versus a mark that promotes transcription^24^, does not recognize that the WIN site is only required for the catalytic activity of one of five MLL/SET enzymes^11,25^, and cannot explain how WIN site inhibitors induce transcriptional changes without altering H3K4me^11^.

We reasoned that comparison of the impact of WDR5 loss versus WIN site inhibition could resolve whether WIN site inhibitors block some or all of the function of WDR5, and would be timely given that the arsenal of WDR5 inhibitors has recently moved beyond those targeting the WIN site to those that trigger WDR5 degradation^26,27^. We also reasoned that monitoring the impact of WDR5 loss on H3K4me3 and transcriptional patterns would allow us to test the idea that WDR5 regulates transcription via modulation of H3K4 methylation. Here, we report that WIN site inhibitor impacts only a subset of transcriptional events controlled by WDR5, and show that the influence of WDR5 on transcription is not explained via its role in H3K4 methylation. These findings reveal that WDR5 loss cannot be used to model the effects of WIN site inhibitors on cancer cells, show how an essential protein can be partially inhibited to induce a pro-therapeutic response, and support distinct applications of WIN site inhibitors and WDR5 degraders in the clinic.

## Results and discussion

### Comparing WDR5 loss with WIN site inhibition

**Figure 1:**
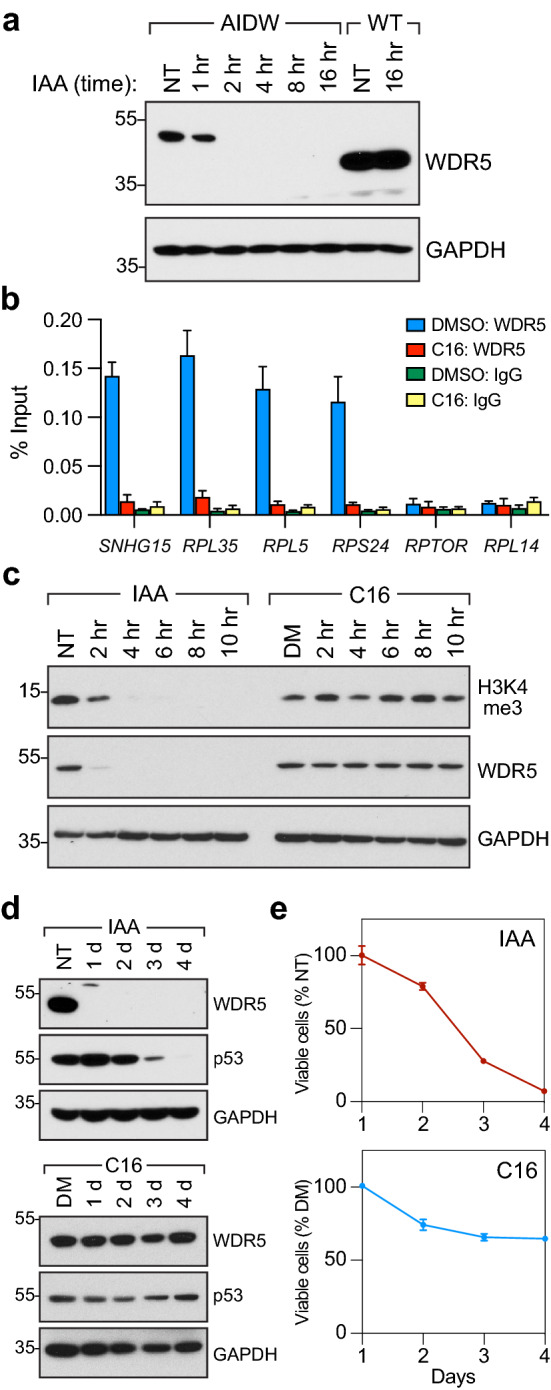
A system to compare loss of WDR5 with WIN site inhibition. (**a**) Wild-type (WT) Ramos cells, or AIDW Ramos cells, were not treated (NT) or treated for the indicated times with 100 μM IAA. WDR5 and GAPDH levels were determined by Western blotting. N = 3. (**b**) AIDW cells were treated with DMSO or 500 nM C16 for 18 h, and ChIP performed with an α-WDR5 antibody or IgG control. Co-precipitating DNAs corresponding to the indicated loci were detected by qPCR. *RPTOR* and *RPL14* are not bound by WDR5. Error bars are standard error. N = 3. (**c**) AIDW cells were treated with 100 μM IAA or 500 nM C16 for the indicated times and H3K4me3, WDR5, and GAPDH levels determined by Western blotting. “NT”; not treated. “DM”; DMSO control. N = 3. (**d**) As in (**c**) but treatments were for four days, and blots probed for WDR5, p53, and GAPDH. N = 3. (**e**) AIDW were treated with 100 μM IAA (top) or 500 nM C16 (bottom) for 1 to 4 days, viable cell numbers determined, and expressed as a percentage of the not treated (NT) or DMSO-treated (DM) control cultures. Error bars are standard error. N = 3. For (**a,c,d**) unprocessed blots are presented in Supplementary Fig. S5.

To ask whether WIN site inhibition impacts some or all of the functions of WDR5, we compared the effects of acute depletion of WDR5 with those of WIN site inhibition in Ramos cells, a Burkitt lymphoma line harboring a *c-MYC* translocation and expressing mutant p53^28^. Because both WDR5 loss^29^ and WIN site inhibition^2,11^ elicit a cellular response via induction of p53, we reasoned that the mutant *TP53* status of Ramos cells would allow us to monitor transcriptional changes independent of complications from p53 activation. We engineered Ramos cells so that WDR5 could be degraded via an auxin-inducible degron (AID)^30^ (Supplementary Fig. S1), creating the line we refer to as “AIDW”. Levels of WDR5 are lower in AIDW cells than the parental Ramos line (Fig. 1a), perhaps due to leaky degradation via the AID tag^31^ or the impact of the genomic modification on *WDR5* transcription. Regardless, upon addition of indole-3-acetic acid (IAA), tagged WDR5 is rapidly depleted from AIDW cells, but not wild-type (WT) Ramos cells (Fig. 1a). To inhibit the WIN site, we treated AIDW cells with 500 nM of WIN site inhibitor C16^12^; a concentration that maximally evicts WDR5 from chromatin (Fig. 1b). In short term treatments, degradation of WDR5 leads to depletion of bulk H3K4me3 (Fig. 1c), consistent with the short half-life of this modified histone^32^. WIN site inhibition, in contrast, has no detectable effect on H3K4me3 levels in this timeframe (Fig. 1c). In longer term treatments, depletion of WDR5 (Fig. 1d) leads to a decrease in cell proliferation, and by day four the number of AIDW cells is less than 10% that of untreated controls (Fig. 1e). Growth of unmodified Ramos cells is not impacted by IAA treatment (Supplementary Fig. S1), demonstrating that this growth deficit is due to AID tagging of WDR5. A 4 day treatment with 500 nM C16, in contrast, reduces viable AIDW cells to just 65% of DMSO-treated controls (Fig. 1e), and the IC_50_ for C16 in these cells is tenfold higher than that required to evict WDR5 from chromatin (Supplementary Fig. S1); likely due to the mutant p53 status of this line. Neither WDR5 degradation nor WIN site inhibition results in changes in the distribution of cell cycle phases (Supplementary Fig. S1). From this analysis, we conclude that AIDW cells are suitable for evaluating the consequences of loss of WDR5 on H3K4me3 and transcriptional processes, and for comparing degradation of WDR5 with selective inhibition of the WIN site. We also conclude that displacement of WDR5 from chromatin by C16 does not recapitulate the effects of loss of WDR5 on Ramos cell H3K4me3 levels or viability.

### WIN site inhibitor induces a subset of transcript changes caused by WDR5 loss

**Figure 2:**
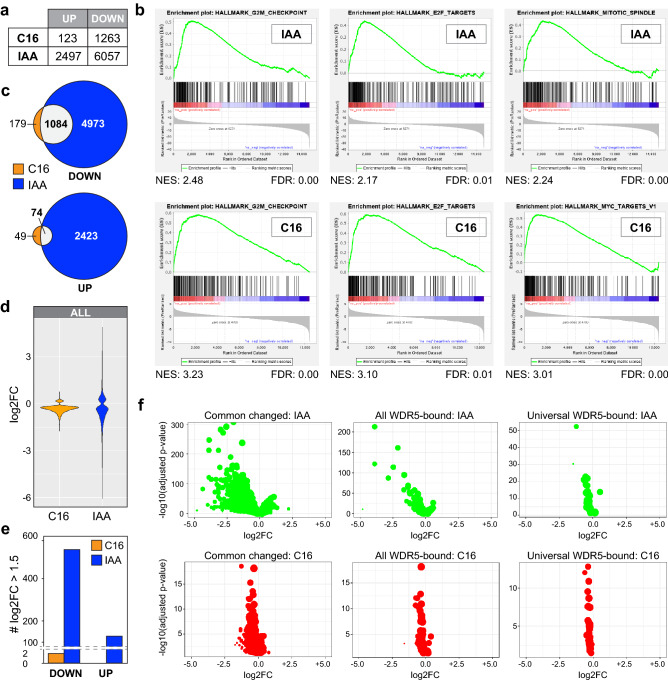
Transcriptomic impact of WDR5 loss versus WIN site inhibition. (**a**) Summary of RNA-Seq, showing the number of differentially expressed genes (FDR < 0.05) altered by 18 h treatment of AIDW cells with 500 nM C16 or 100 μM IAA. N = 4. (**b**) Gene set enrichment analysis (GSEA) of RNA-Seq data from IAA-(top) and C16-(bottom) treated AIDW cells. Top three categories are shown; full results are in Supplementary Tables S1 and S2. *NES* normalized enrichment score, *FDR* false discovery rate. (**c**) Venn diagram, showing the overlap of transcripts decreased or increased in response to IAA or C16 treatment of AIDW cells. (**d**) Violin plot, displaying the magnitude of significant transcript changes associated with C16 or IAA treatment, plotted as log_2_-fold change (log2FC). (**e**) Graph comparing the number of transcripts with log2FC > 1.5 in AIDW cells in response to IAA or C16 treatment. (**f**) Volcano plots, comparing transcript changes in IAA- (top) or C16- (bottom) treated AIDW cells, confined to either: (i) transcripts that change in both the IAA and C16 samples (left), (ii) transcripts from genes bound by WDR5 in Ramos cells^4^ (middle), and (iii) transcripts from genes “universally” bound by WDR5 in human cell lines^2^ (right). Bubble size indicates mean of normalized counts of all samples, normalizing for sequencing depth.

Next, we compared the effects of WDR5 degradation and WIN site inhibition on the transcriptome of AIDW cells after 18 h of treatment with IAA or C16 (Supplementary Fig. S2). Five major observations were made: (i) loss of WDR5 has a more pronounced impact on the transcriptome than WIN site inhibition, as judged by principal component analysis (Supplementary Fig. S2) and by the number of genes dysregulated (Fig. 2a); (ii) both treatments share similarities in the types of genes dysregulated, particularly those relating to cell cycle processes and protein synthesis (Fig. 2b, Supplementary Fig. S2, Supplementary Tables S1, S2); (iii) transcript changes caused by WIN site inhibition are largely a subset of those caused by WDR5 depletion (Fig. 2c, Supplementary Fig. S2); (iv) gene expression changes unique to WDR5 degradation are enriched in those linked to mitochondrial processes (Supplementary Fig. S2); and (v) WDR5 degradation results in larger changes in transcript levels than WIN site inhibition (Fig. 2d) with ~ 550 transcripts altered by > log_2_1.5-fold in IAA-treated cells, compared to just two with C16 (Fig. 2e). The greater impact of WDR5 loss on transcript changes is observed genome-wide (Fig. 2d), at genes that respond to both IAA and C16, and at genes bound by WDR5 in Ramos cells^4^ (Fig. 2f). It is not observed, however, at genes “universally” bound by WDR5 in human cell lines^2^ (Supplementary Table S3), revealing that these genes—most of which encode ribosomal proteins (Supplementary Fig. S2) and two thirds of which are shared MYC–WDR5 targets^4^ (Supplementary Table S3)—are some of the few sites in the genome where the impact of WDR5 loss and WIN site inhibition are comparable.

Based on these data, we conclude that depletion of WDR5 has a widespread effect on the transcriptome; one that is much broader that that elicited by WIN site inhibition, both in terms of the number of genes dysregulated and the magnitude of the transcript changes. We further conclude that most transcript changes caused by WIN site inhibition are a subset of those caused by WDR5 depletion, indicating that transcriptional responses to C16—which are biologically focused on genes connected to protein synthesis and the cell cycle—are mediated via an on-target mechanism. Moreover, because there are only ~ 400 genes that are bound by WDR5 in Ramos cells^4^, we also conclude that a majority of transcript changes resulting from WDR5 depletion occur at sites where physical recruitment of WDR5 to chromatin is not detected.

### WDR5 impacts transcriptional patterns independent of H3K4me3

The role of H3K4me3 as activating epigenetic mark is controversial^24^. Nonetheless, because H3K4me3 is widely posited to be the mechanistic focal point of WDR5 function—and thus of WDR5 inhibitors; e.g.^22,33–39^)—and to attempt to understand the extensive transcriptomic impact of WDR5 depletion—we next asked whether mRNA changes caused by loss of WDR5 correlate with changes in H3K4me3 status.

**Figure 3:**
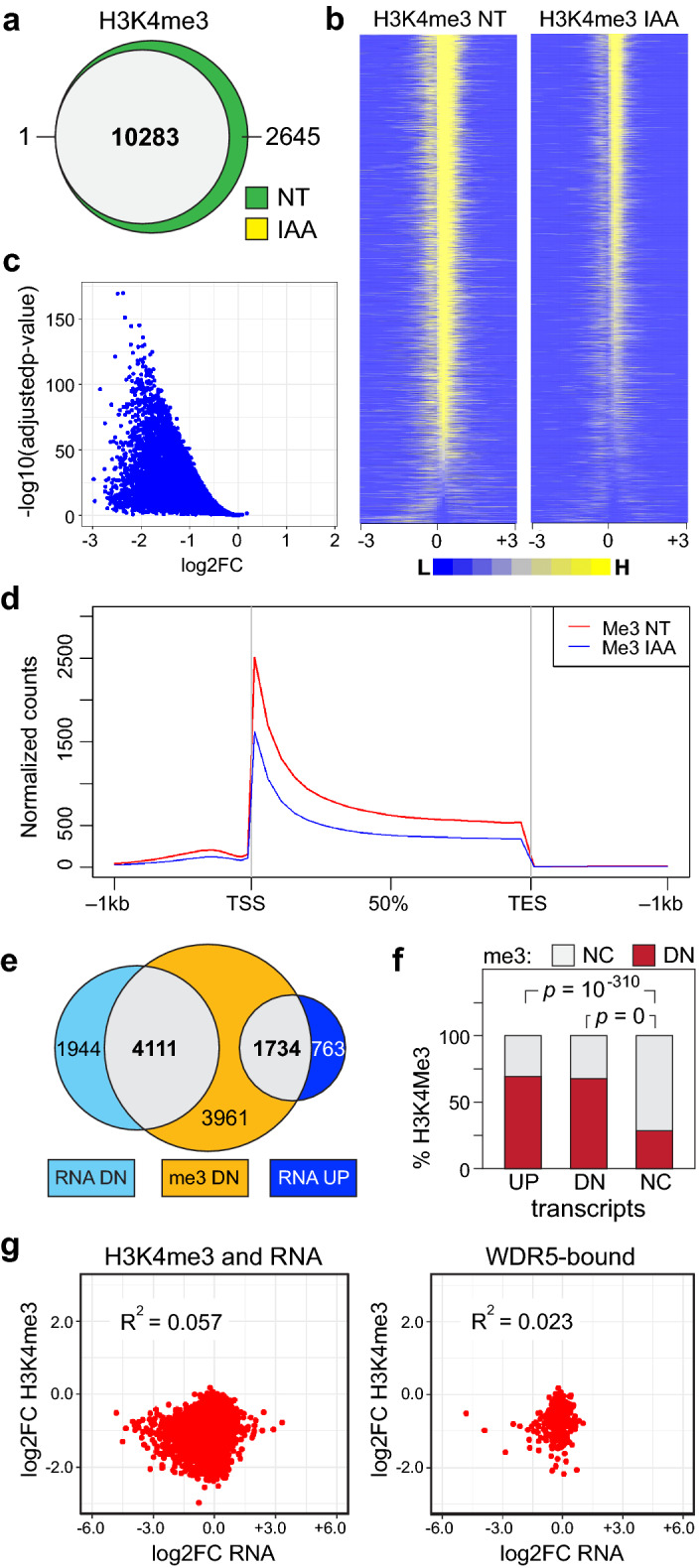
Impact of WDR5 degradation on chromatin-associated H3K4me3. (**a**) Venn diagram, displaying the overlap of H3K4me3 ChIP-Seq peaks in non-treated (NT) or treated (18 h/100 μM IAA) AIDW cells. N = 3 for each set. (**b**) Heatmaps of the average normalized peak intensity (100 bp bins) for H3K4me3 peaks in NT and IAA-treated AIDW cells. Peaks are ranked according to the NT sample. Included are regions 3 kb upstream (− 3) and downstream (+ 3) of the peak zenith (0). (**c**) Volcano plot, comparing the log2FC in H3K4me3 peak intensity (IAA versus NT) against the − log10(adjusted p-value). (**d**) Averaged H3K4me3 peak shape and distribution in NT and IAA-treated AIDW cells, relative to the transcription start site (TSS), the transcription end site (TES), and 1 kb upstream and downstream of each. (**e**) Venn diagram, displaying the relationship between genes with decreased H3K4me3 and transcripts displaying significant decreases or increases in IAA-treated versus NT cells. (**f**) Graph showing the percentage of genes with not changed (NC; gray) or decreased (DN; red) H3K4me3 (me3) levels in IAA-treated cells, binned according to whether transcripts for those genes are increased (UP), decreased (DN), or not changed (NC) by IAA-treatment in RNA-Seq. *p*-values for UP against NC and DN against NC are shown; the *p*-value for UP against DN is 0.1738. (**g**) Scatter plots comparing log2FC in H3K4me3 versus RNA for IAA-treated AIDW cells. Comparisons are for (left) all genes that have a measurable H3K4me3 peak and a measurable transcript, and (right) genes that are bound by WDR5 in Ramos cells^4^ and have measurable H3K4me3 and RNA signals. The coefficient of determination (R^2^) is shown inside each plot.

We used chromatin immunoprecipitation sequencing (ChIP-Seq) to localize H3K4me3 on chromatin in AIDW cells, before and after WDR5 depletion. In unperturbed cells, we tracked ~ 12,500 sites of H3K4me3 (Supplementary Fig. S3), the majority of which are within 5 kb of an annotated transcription start site, and show the expected distribution^40^, peaking immediately downstream of the TSS (Supplementary Fig. S3). Despite the disappearance of bulk H3K4me3 in western blotting (Fig. 1c), ~ 10,300 sites of H3K4me3 are still detected by ChIP-Seq after 18 h of IAA treatment (Fig. 3a, Supplementary Fig. S3), albeit at reduced levels (Fig. 3b,c). Similar differences between bulk histone modifications and those detected by ChIP-Seq have been reported after treatment of cells with EZH2 inhibitors^41,42^, and could be due to different sensitivities of the two factors (western blotting versus ChIP), different pools of modified histones, or other method-specific considerations. Reductions in H3K4me3 occur throughout the transcription unit (Fig. 3d), and are most pronounced at genes with the lowest levels of H3K4me3 (Supplementary Fig. S3). Overlaying these data with transcriptomic changes (Supplementary Fig. S3), we observe that although many genes suppressed by WDR5 depletion experience decreased H3K4me3, there is no trend between methylation and transcript changes (Fig. 3e). Forty percent of genes undergoing a reduction in H3K4me3 are transcriptionally non-responsive, and more than two thirds of the genes induced by WDR5 depletion display decreased H3K4me3 levels. Indeed, both induced and suppressed genes are more likely to experience a decrease in H3K4me3 than those that are transcriptionally unresponsive (Fig. 3f), and the correlation between H3K4me3 and transcript changes is poor (Fig. 3g)—and does not improve if restricted to genes that change in response to both IAA and C16 (Supplementary Fig. S3), genes bound by WDR5 in Ramos cells (Fig. 3g), or universal WDR5-bound genes (Supplementary Fig. S3).

Taken together, these data establish that WDR5 depletion leads to a genome-wide reduction in H3K4me3, with weakly methylated sites being most vulnerable to WDR5 loss, and show there is little if any correlation between the effects of WDR5 depletion on H3K4me3 and steady-state transcript levels. Although consistent with the idea that WDR5 is core part of the MLL/SET complexes that deposit H3K4me3^1^, these findings are inconsistent with the idea that the transcriptional influence of WDR5 in this setting is a result of its H3K4me3 writer functions.

### WDR5 depletion rapidly alters transcription at thousands of genes

The broad impact of WDR5 depletion on the steady-state transcriptome suggests that many of the transcript changes resulting from degradation of WDR5 are indirect, perhaps caused by secondary responses to a more limited set of primary transcriptional events. To identify high-confidence primary transcriptional targets of WDR5, therefore, we used the global nuclear run on method PRO-Seq^43^ to visualize how transcription is altered across the genome soon after WDR5 depletion—2 and 4 h following addition of IAA to AIDW cells.

**Figure 4:**
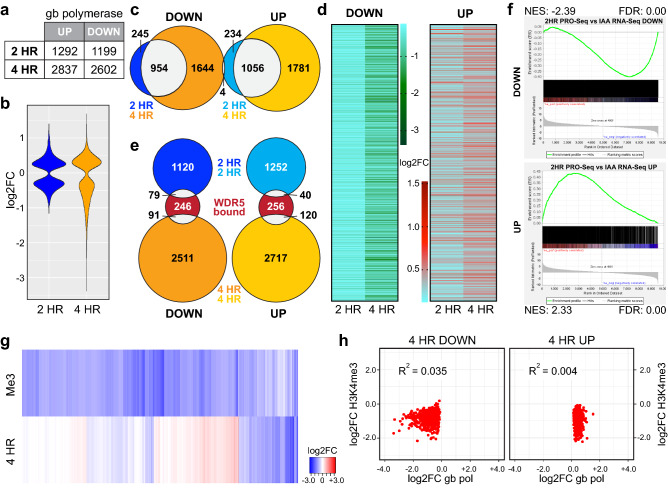
Rapid impact of WDR5 loss on transcription. (**a**) Summary of PRO-Seq from AIDW cells treated with 100 μM IAA for 2 or 4 h. Table shows the number of genes that had a significant increase or decrease in gene body (gb)-associated RNA polymerases at the 2 and 4 h timepoints (FDR < 0.05). N = 3. (**b**) Violin plot, displaying the magnitude of all significant transcription changes associated with 2 or 4 h of IAA treatment, plotted as log2FC. (**c**) Venn diagram, showing overlap of genes in the 2 and 4 h IAA treatment sets with significant changes in gb-associated polymerases, according to whether gb-polymerase density decreased (DOWN) or increased (UP) with IAA treatment. (**d**) Heatmaps, showing log2FC values for genes with decreased or increased gb-associated polymerases in the 2 and 4 h IAA treatments. (**e**) Venn diagram, showing the overlap of genes with significant changes in gb-associated polymerases with those bound by WDR5 in Ramos cells^4^, broken down according to whether gb-polymerase density decreased (DOWN) or increased (UP) with IAA treatment. (**f**) GSEA showing the enrichment of genes with significant decreases (top) or increases (bottom) in transcript changes detected in IAA RNA-Seq against gb-associated polymerases following 2 h of IAA treatment (PRO-Seq). *NES* normalized enrichment score, *FDR* false discovery rate. (**g**) Heatmap, showing log2FC values for genes changed in either gb-associated polymerases in 4 h IAA treatments (PRO-Seq) or H3K4me3 (me3) levels in IAA-treated cells. (**h**) Scatter plots, comparing log2FC in H3K4me3 induced by WDR5 degradation with log2FC in gb-associated polymerases induced by WDR5 degradation after 4 h (4 HR) of IAA treatment. The plot on the left shows genes with decreased polymerase density; the plot on the right shows genes with increased polymerase density. The coefficient of determination (R^2^) is shown.

WDR5 depletion rapidly causes changes in gene body-associated polymerases at thousands of genes (Supplementary Table S4). Four hour treatment dysregulates more genes (Fig. 4a) and to a greater extent (Fig. 4b) than two hours, although a majority of genes that respond at 2 h show persistent (Fig. 4c) and progressive (Fig. 4d, Supplementary Fig. S4) responses at 4 h. Notably, most genes bound by WDR5 in Ramos cells (Fig. 4e), and most universal WDR5-bound loci (Supplementary Fig. S4), do not respond to WDR5 loss. Overall, there is a high degree of congruence between the early transcriptional changes induced by WDR5 depletion and later changes in the transcriptome (Fig. 4f, Supplementary Fig. S4, Supplementary Table S5): Seventy percent of genes with decreased transcription at 2 and 4 h have decreased transcript levels at 18 h (Supplementary Fig. S4). Notably, suppression of genes connected to mitochondria (Supplementary Fig. S2), detected by RNA-Seq at 18 h, are not evident in the PRO-Seq data, suggesting that these genes are suppressed as a secondary consequence of WDR5 degradation, perhaps in response to decreased energy requirements caused by decreased cell proliferation (Fig. 1e). For induced genes, the overlap between genes altered in the PRO-Seq and RNA-Seq is just 40%, suggesting that a portion of the increased transcription—which curiously occurs on both the sense and anti-sense strands (Supplementary Fig. S4)—is non-productive. Particularly for suppressed genes, therefore, much of the impact of WDR5 degradation on the transcriptome results from early and direct effects at the level of gene transcription.

Finally, we asked if changes in transcription caused by WDR5 depletion correlate with changes in H3K4me3. As we observed in our transcriptome analysis, there is little connection between the two (Fig. 4g). One quarter of genes with altered transcription show no subsequent H3K4me3 change, as many of the induced as the suppressed genes have decreased H3K4me3 at 18 h, and correlations between transcript and H3K4me3 changes are poor (Fig. 4h, Supplementary Fig. S4h–i). Just as steady-state transcripts fail to explain H3K4me3 changes, therefore, primary transcriptional events fail to predict how H3K4me3 will change in response to WDR5 depletion in Ramos cells.

In total, this analysis demonstrates that WDR5 is a bonafide regulator of transcription of thousands of genes, and that a significant percentage of steady-state transcript changes caused by WDR5 depletion result from direct changes in transcription—particularly for suppressed genes. The broad effects of WDR5 loss on transcription contrast strongly with the effects of WIN site inhibition, which alters transcription at tenfold fewer genes^2,11^ and has a significantly more limited impact on global transcript patterns (Fig. 2). This analysis also reveals an unexpected role of WDR5 in suppression of antisense transcription, and it further supports our contention that a majority of transcriptional events under the control of WDR5 are independent of its role in depositing H3K4me3. Further work will be required to determine which of the many functions of WDR5, independent of H3K4me3, are responsible for its broad impact on transcriptional processes in this setting.

## Conclusions

In terms of applying WDR5 inhibitors as a cancer therapy, our work has three important ramifications. First, its supports the idea that WDR5 impacts transcription in a manner disconnected from changes in H3K4 methylation. As such, it is unlikely that H3K4me3 is the route through which WDR5 inhibitors or degraders drive transcriptional changes. Second, our work demonstrates that WIN site inhibitors affect only a subset of WDR5 function in gene expression. This finding provides a rationale for how an effective therapeutic window for WIN site inhibitors can be obtained for a pan-essential protein like WDR5, because these inhibitors disable only part of the functional repertoire of WDR5. This finding further suggests that clinical application of WIN site inhibitors, and determination of their mechanism of action, should not be guided by loss of WDR5-based approaches. Finally, our work implies that future WIN site inhibitors and WDR5 degraders will each require unique validation and biomarker strategies, and will most likely have disparate clinical applications.

## Methods

### Plasmids

pX330-U6-Chimeric_BB-CBh-hSpCas9 was a gift from Feng Zhang (Addgene #42230). pUC57, containing a U6 promoter, scaffold, and terminator was synthesized by Genscript and gRNAs for WDR5 (CTTCAGTGTTCGGGGTCGGA and TTATTTACTGACCGCATATC) individually inserted by whole plasmid PCR. The AID-WDR5 targeting vector was made by assembly of six fragments into pBluescript II SK+: (i) [WDR5 Promoter region (Chr9: 134,138,834–134,139,883) as KpnI/XhoI]; (ii) [AID Tag : Ex2-Int2 (CHr9: 134,139,881–134,140,009): loxP: Int2-Ex3 (Chr9: 134,140,503–134,140,708) was synthesized by Genscript and inserted as XhoI/HindIII]; (iii) [Puromycin resistance (from pcDNA3.1-Puro) as HindIII/XmaI]; (iv) P2A (synthesized by Genscript) as XmaI/EcoRI]; (v) [Ex2-Int2 (Chr9: 134,139,881–134,140,009): loxP as EcoRI/BamHI]; and (vi) [Int2-Ex3-Int3 (Chr9: 134,140,336–134,141,445) as BamHI/XbaI]. pLL4-osTIR1 was made by PCR-amplification of osTIR1-9xMyc from pBabe-osTIR1-9Myc (gift from Don Cleveland, Addgene plasmid # 47328) and used to replace GFP in pLentiLox (gift from Al Reynolds).

### Cell culture

Ramos cells were obtained from the ATCC (CRL-1596) and cultured in RPMI media supplemented with l-glutamine, 10% FBS, 100 IU/ml Penicillin, and 100 μg/ml streptomycin. Ramos cells expressing CRE–ERT2 were described previously^4^ and used to construct the AIDW line. AIDW cells were created in a four step process. First, the AID tag was integrated into the endogenous *WDR5* gene via CRISPR-mediated homologous recombination by electroporation with 10 μg targeting vector, 15 μg of gRNA vector, and 15 μg of pX330-U6-Chimeric_BB-CBh-hSpCas9. Second, after a 2-day recovery, stable cells were selected with 200 ng/ml puromycin, expanded, treated with 4-OHT to excise the puromycin cassette, and clones obtained by limited dilution. These clones were then expanded and screened by Western blotting to identify clones in which the apparent molecular weight of WDR5 was shifted by the expected amount for the AID tag. At this stage, positive clones we recovered expressed both AID-tagged and untagged WDR5 species. Third, to target the remaining *WDR5* allele, steps one and two were repeated with a second unique gRNA. Finally, a clone expressing only AID-tagged WDR5 was transduced to express the OsTIR1 ubiquitin ligase carrying a c-Myc epitope tag. Lentiviral infections of the selected Ramos cell clone with pLL4-osTIR1 were performed as described^3^; OsTIR1 expression was confirmed by Western blotting with antibodies against the Myc epitope.

### Cell proliferation and cell cycle analysis

For WDR5 degradation, cells were treated with 100 μM IAA (Sigma-Aldrich). For WIN site inhibition, cells were treated with 500 nM C16^12^ or 0.1% DMSO (control). To compare the effects of WDR5 degradation with WIN site inhibition on AIDW cell proliferation, AIDW cells were seeded in 96-well plates and treated for up to four days with either 0.1% DMSO, 100 uM IAA, or 500 nM C16. At each timepoint, viable cells were quantified using Promega CellTiter-Glo Reagent. For cell cycle analysis, 10^6^ AIDW cells were collected after no treatment, or treatment with 0.1% DMSO, IAA, or C16, fixed in ice-cold 70% ethanol, and stored at − 20 °C for at least 4 h. Fixed cells were washed with 1× phosphate buffered saline (PBS), resuspended in propidium iodide (PI) staining buffer (1× PBS + 10 μg/ml PI + 100 μg/ml RNAse A + 2 mM MgCl_2_) and stained overnight at 4 °C. Cell cycle distribution was quantified using a Becton Dickinson LSRFortessa instrument and BD FACSDiva software. At least 10,000 cells were counted using forward and side scatter pulse geometry gating to select single cells for each sample. IC_50_ determination for C16 in AIDW cells was performed as described^11^.

### Western blotting

Whole cell lysates were prepared, separated by SDS-PAGE, and probed by Western blotting as described^4,44^. Antibodies used were: α-WDR5 #13105, α-H3K4me3 #9751, α-GAPDH–HRP #8884 and #5174, α-p53 #32532, α-Myc #2278 (Cell Signaling); α-p53 (Santa Cruz sc-126); α-rabbit Fc Secondary 31463 (ThermoFisher); α-mouse Fc Secondary Antibody and α-rabbit IgG–HRP, Light Chain Specific 211-032-171 (Jackson ImmunoResearch).

### Chromatin immunoprecipitation

ChIP was performed as described^4^ using antibodies against WDR5 (#13105) or a rabbit IgG control (#2729; Cell Signaling). ChIP-qPCR primers used were:

- *SNHG15:* (CGCCACTGAACCCAATCC and TCTAGTCATCCACCGCCATC),
- *RPL35:* (CTTGTGCAGCAATGGTGAGA and GCCTAGGTGGCAGATAGAATC),
- *RPL5:* (CCTGCAGGTCTCTGTCGAG and GGCATACGGGCAAGAAAAG),
- *RPS24*: (TTGGCTGTCTGAAGATAGATCG and CGCGTGCCTATAGCTCAAGT),
- *RPTOR:* (CCCTTGAGCAGATGAATACT and GACAATTTGCAGGACAGAG),
- *RPL14:* (GTCTCCTTTGGACCTCATGC and ATGGCCTGTCTCCTCACTTG).

ChIP signals were expressed as percent input. For ChIP-Seq, ChIP was performed with an anti-H3K4me3 antibody (PA5-27029; Thermo Fisher). ChIP DNA was purified and libraries prepared as described^4^. Libraries were sequenced on an Illumina NovaSeq 6000 instrument by the VANTAGE Core at Vanderbilt University.

### RNA-sequencing and precision run on-sequencing

For RNA-Seq, cells were either not treated, or treated for 18 h with 0.1% DMSO, 100 μM IAA, or 500 nM C16, for 18 h. They were then collected in Trizol (Invitrogen), RNA purified^45^, and submitted to the VANTAGE Core who performed ribosomal RNA depletion, library preparation, and sequencing on an Illumina NovaSeq 6000. For PRO-Seq, 3 × 10^7^ cells were either untreated or treated with IAA 100 μM IAA for 2 or 4 hours, at which point cells were harvested and PRO-Seq reactions performed as described^46,47^. PRO-Seq libraries were submitted for sequencing on an Illumina NovaSeq 6000 with 150 paired-end reads by the VANTAGE Core.

### Bioinformatics analyses

(1) *ChIP-Seq* ChIP-Seq reads were aligned to the human genome hg19 using Bowtie2^48^. Peaks in each sample were called using MACS2 with q-value of 1e−5^49^. Peaks were annotated using Homer (http://homer.ucsd.edu/homer/) to assign target genes. Consensus peaks in each condition were identified using DiffBind^50^. Differential analyses were performed by DESeq2^51^. False Discovery Rate (FDR) < 0.05 was used to identify significantly changed peaks. (2) *RNA-Seq* After trimming by Cutadapt^52^, RNA-Seq reads were aligned to hg19 using STAR^53^ and quantified by featureCounts^54^. Differential analysis was performed by DESeq2^51^. FDR < 0.05 was used to identify significantly changed genes. (3) *PRO-Seq* After adapter trimming and low quality sequence removal by Cutadapt^52^, PRO-seq reads longer than 15 bp were reversed complemented using FASTX-Toolkit (http://hannonlab.cshl.edu/fastx_toolkit). Reverse-complemented reads were aligned to hg19 using Bowtie2m^48^. Reads mapping to rRNA loci and reads with mapping quality < 10 were removed. Reads were normalized by the RLE implemented in DESeq2^48^. Alignment files were used as inputs to NRSA (http://bioinfo.vanderbilt.edu/NRSA/) for estimating alterations of RNA polymerase abundance in proximal-promoter and gene body regions^55^. The promoter-proximal region was defined by examining each 50 bp window with a 5 bp sliding step along the coding strand spanning ± 500 bp from known TSSs; the 50 bp region with the largest number of reads was considered as the promoter-proximal region and its read density was calculated^56^. Gene body was defined as the region from + 1 kb downstream of a transcription start site (TSS) to its transcription termination site. DESeq2^51^ was implemented to detect significant transcriptional changes for promoter-proximal and gene body regions accounting for the batch effect. Transcriptional changes with an FDR < 0.05 were considered significant.

## Supplementary Information

Supplementary Figures.

Supplementary Table S1.

Supplementary Table S2.

Supplementary Table S3.

Supplementary Table S4.

Supplementary Table S5.

## Data Availability

Genomic data have been deposited at the Gene Expression Omnibus (GEO; GSE183781).
