# Supplementary material for: WIN site inhibition disrupts a subset of WDR5 function
Source: Sci Rep. 2022 Feb 3;12:1848. doi: 10.1038/s41598-022-05947-9 (PMC8813994; doi:10.1038/s41598-022-05947-9)
Supplement: Supplementary file 1 — Supplementary Figures. [file 41598_2022_5947_MOESM1_ESM.pdf]

Supplementary Material for "WIN site inhibition disrupts a subset of WDR5 function" by Andrew J. Siladi, Jing Wang, Andrea C. Florian, Lance R. Thomas, Joy H. Creighton, Brittany K. Matlock, David K. Flaherty, Shelly L. Lorey, Gregory C. Howard, Stephen W. Fesik, April M. Weissmiller, Qi Liu, and William P. Tansey

Contents:

Supplementary Figure legends
List of Supplementary Tables
Supplementary Figures S1–5

Supplementary Figure Legends

Supplementary Fig. S1. A system to compare WDR5 loss versus WIN site inhibition. **a.** Strategy for tagging endogenous *WDR5* loci with the AID tag. Top panel shows a depiction of the 5' region of the human *WDR5* gene on chromosome 9q34.2. Exons are numbered and depicted as boxes; coding sequences are represented as blue; non-coding sequences as grey. Splicing events are depicted by blue lines. The location of gRNAs used to trigger cutting by Cas9 are indicated. Middle panel shows the first step in the modification, which is to introduce sequences encoding the AID tag (orange) as well as a puromycin resistance cassette ($PURO^R$), and two loxP sites, as indicated. Introduced sequences also include a P2A "self-cleaving" peptide that permits translation of downstream *WDR5* sequences, separate from those of the AID and $PURO^R$ sequences. The bottom panel shows the locus after excision of the $PURO^R$ cassette by transient activation of CRE recombinase. **b.** Expression of AID-tagged WDR5 and OsTIR1 in AIDW cells. Lysates were prepared from wild-type (WT) Ramos cells, or Ramos cells engineered to express AID-tagged WDR5 and OsTIR1 (AIDW), and WDR5, OsTIR1, or GAPDH (loading control) levels determined by Western blotting. OsTIR1 was detected by antibodies against the Myc epitope tag. **c.** Wild-type Ramos cells were treated with 100 µM IAA for one to four days, viable cell numbers determined, and expressed as a percentage of the not treated (NT; vehicle) control cultures. Error bars are standard error. N = 3. **d.** Dose response of AIDW cells to C16 in a three day treatment paradigm. Data are expressed as the percentage of cells remaining at day three, compared to the DMSO control. N = 3. Error bars are standard error. IC_{50} was calculated by fitting the data to a normalized-response model. **e.** Bar graphs, showing distribution of cell cycle phases as determined by flow cytometry for AIDW Ramos cells treated with (left) 100 µM IAA or not treated (NT), or (right) 500 nM C16 or DMSO, for 18 hours. N = 3. Error bars are standard error. For (**b**) full-length blots are presented in Supplementary Fig. S5.

Supplementary Fig. S2. Comparison of the effects of WDR5 degradation and WIN site inhibition on the transcriptome of AIDW cells. **a.** Z-transformation heatmaps displaying replicates of RNA-Seq analysis of samples from 18 hour C16- or IAA-treated AIDW cells. DMSO is the control for C16; NT (no treatment) is the control for IAA. Transcripts that are significantly (FDR < 0.05) impacted in treated samples are shown. R; replicate number. N = 3. **b.** Principal component analysis (PCA) plot of RNA-seq data based on gene expression levels for the four treatment regimens (DMSO, C16, NT, and IAA). **c.** Gene ontology (GO) term enrichment analysis of transcripts significantly (FDR < 0.05) decreased by IAA-treatment of AIDW cells, compared to NT controls. Only the top eight GO terms (according to fold-enrichment) are shown. The numbers inside each bar are the respective p-values; the numbers to the right of the graph are the number of gene hits in each category. **d.** As in (c) but for transcripts significantly increased by IAA-treatment. **e.** As in (c) but for transcripts significantly (FDR < 0.05) decreased by C16-treatment of AIDW cells, compared to DMSO controls. **f.** As in (d) but for transcripts significantly increased by C16-treatment. Note that for decreased transcripts, IAA and C16 share thematically similar categories relating to protein synthesis; for increased transcripts, IAA and C16 share thematically similar categories relating to mitosis, the cell cycle, and DNA replication/repair. **g.** GO term enrichment analysis of transcripts significantly (FDR < 0.05) decreased by both IAA- and C16-treatment of AIDW cells, compared to their respective controls. Rest of the figure as in (c). Most of the

genes in the last six categories are the same and encode protein subunits of the ribosome. **h.** As in (g) but for transcripts significantly increased by both treatments. **i.** GO term enrichment analysis of transcripts significantly (FDR < 0.05) decreased only in IAA- (and not C16)-treated AIDW cells. Rest of the figure as in (c). These transcripts are strongly connected to nuclear encoded mitochondrial processes. **j.** As in (i) but for transcripts significantly increased only in IAA-treated AIDW Ramos cells. For (c–j) the GO domain used is "Biological Process" (BP). **k**. Heatmap, displaying $\log_2$ fold change (log2FC) in transcripts (IAA, top; C16, bottom) that altered in both treatments and confined to the set of genes "universally" bound by WDR5 in human cell lines. Although there are ~100 universal WDR5-bound genes, only 43 respond to WDR5 perturbation at the transcriptional level. Genes labeled in red cluster under the GO BP (GO:0006412) term "translation" and most are ribosomal protein genes.

Supplementary Fig. S3. Effects of WDR5 degradation on genome-wide distribution of H3K4me3 in AIDW Ramos cells. **a.** Venn diagram, displaying the relationship of peaks of H3K4me3 identified in non-treated (NT) AIDW cells in each of three replicates (R1–R3), as determined by ChIP-Seq. N = 3. **b.** Distribution of H3K4me3 binding sites in NT AIDW cells, relative to an annotated transcription start site (TSS). ChIP-Seq peaks are plotted according to distance from nearest TSS (kb), binned by region. On the x-axis, blue numbers represent bins upstream of the TSS; red numbers represent bins downstream of the TSS. The y-axis shows the percentage of each bin, relative to all peaks; the numbers above each bar show the number of peaks in each bin. **c.** Averaged H3K4me3 peak shape and distribution in NT AIDW cells, relative to the TSS, the transcription end site (TES), the midpoint of the transcription unit (50%), and 1 kb upstream of the TSS and downstream of the TES. **d.** Venn diagram, displaying the number of peaks of H3K4me3 identified in AIDW cells treated for 18 hours with IAA in each of three replicates (R1–R3), as determined by ChIP-Seq. N = 3. **e.** Boxplot, showing the $\log_2$ fold-change (log2FC) of H3K4me3 signal (IAA versus NT), binned according to the strength of the H3K4me3 signal in NT cells. "<1Q" are H3K4me3 sites in the first quartile, "1Q–M" are sites between the first quartile and the median, "M–Q3" are sites between the median and the third quartile, and ">Q3" are sites in the fourth quartile. For each quartile, boxes and whiskers represent the distribution of log2FC of H3K4me3 signal based on a five number summary (Q1-1.5*IQR, first quartile (Q1), median, third quartile (Q3), and Q3+1.5*IQR). Outliers are shown as circles. IQR (interquartile range): Q1 to Q3. **f.** Seven IGV screenshots of representative ChIP-Seq data for H3K4me3 in NT (red) and IAA-treated (blue) AIDW Ramos cells. The panels are organized by increasing peak intensity of H3K4me3 in NT cells, and each shows a 5 kb chromosome segment. Note that the data range (square brackets) is different in each panel, but identical for the NT and IAA samples within each panel. **g**. Heatmap, showing $\log_2$ fold-change (log2FC) values for genes changed in either transcript levels (RNA-Seq) or H3K4me3 (me3 ChIP-Seq) levels in IAA-treated cells. **h.** Scatter plots, comparing $\log_2$ fold-change (log2FC) in H3K4me3 versus RNA for linked genes, induced by IAA-treatment of AIDW cells. Comparisons are confined to (top) transcripts from genes that change in both the IAA and C16 RNA-Seq samples or (bottom) transcripts from genes "universally" bound by WDR5 in human cell lines [12]. The coefficient of determination (R^2) is shown inside each plot.

Supplementary Fig. S4. Effects of WDR5 degradation on genome-wide transcription in AIDW cells. **a.** Heatmaps showing RNA polymerase distribution within 5 kb (K) of a TSS for genes that show

significant gene body polymerase changes with both two and four hours of IAA treatment, as determined by PRO-Seq. Genes with decreased polymerase density are on the left heatmap; those with increased polymerase density are on the right. For each heatmap, genes are ordered by promoter-proximal RNA polymerase density in the respective 2 HR set. **b.** Scatter dot plots showing log_2-fold changes (log2FC) for genes with altered gene body associated RNA polymerase at both the two and four hour IAA timepoints. Genes with decreased polymerase density in the gene body are on the left; those with increased polymerase density are on the right. The black horizontal lines through indicate the mean of the data. **c.** Venn diagram, showing the overlap of genes with significant changes in gene body-associated polymerases with those "universally" bound by WDR5 in human cell lines, and broken down according to whether active polymerase density decreased (gene body down; DOWN) or increased (gene body up; UP) with IAA treatment at the two (top) or four (bottom) hour timepoints. **d.** GSEA showing the enrichment of genes with significant decreases (top) or increases (bottom) in transcript changes detected in IAA RNA-Seq against gb-associated polymerases following four hours of IAA treatment (PRO-Seq). NES; normalized enrichment score. FDR; false discovery rate. **e.** Results of gene set enrichment analysis (GSEA) of PRO-Seq data from IAA-treated AIDW cells at the two (2 HR; top row) and four (4 HR; bottom row) timepoints. Full GSEA results are in Supplementary Table S5. NES; normalized enrichment score. FDR; false discovery rate. Note the similarities between the two and four hour timepoints. **f.** Venn diagrams, showing the overlap of genes with significant changes in gene body-associated polymerases with those transcripts that change at 18 hours of IAA treatment, as determined by RNA-Seq. Diagrams are organized according to whether active polymerase density decreased (gene body down; DOWN) or increased (gene body up; UP) with IAA treatment at the two (4 HR; top) or four (4 HR; bottom) hour timepoints. **g.** Normalized read counts of PRO-Seq signals plotted as a function of distance (bp) upstream and downstream of the TSS. Plots on the left are for those genes with decreased polymerase density (gene body down) at both the two (IAA_2 HR) and four (IAA_4 HR) treatments, compared to the no-treated (NT) control; plots on the right are for those with increased polymerase density (gene body up). Plots at the top show polymerases transcribing the sense strand; plots at the bottom show antisense transcription. For genes with decreased transcription, changes at two hours primarily occur on polymerases within gene bodies, whereas most additional changes going from two to four hours are within the promoter-proximal polymerase peaks (both sense and antisense). Genes with increased transcription, in contrast, show increases promoter-proximal and gene body-associated polymerases at the two hour timepoint, both of which further increase by four hours. Note that loss of WDR5 also leads to an increase in levels of RNA polymerases active on the antisense strand—both upstream and downstream of the TSS. **h.** Venn diagram, displaying the overlap of genes with decreased H3K4me3 (H3K4me3 DOWN) after 18 hours of IAA treatment with genes with decreased or increased polymerase density in the 4 HR PRO-Seq data. **i.** Scatter plots, comparing log_2 fold-change (log2FC) in H3K4me3 induced by WDR5 degradation with log2FC in gene body-associated polymerases (gb pol) induced by WDR5 degradation after two hours (2 HR) of IAA treatment. The plot on the left shows genes with decreased (DOWN) polymerase density; the plot on the right shows genes with increased (UP) polymerase density. The coefficient of determination (R^2) is shown inside each plot.

Supplementary Fig. S5. Unprocessed images for Western blots presented in Fig. 1 and Supplementary Fig. S1.

List of Supplementary Tables (provided separately as Excel files):

Supplementary Table S1. GSEA of RNA-Seq data from AIDW cells treated with IAA.
Supplementary Table S2. GSEA of RNA-Seq data from AIDW cells treated with C16.
Supplementary Table S3. Impact of C16 or IAA on transcript levels from "universal" WDR5-bound genes.
Supplementary Table S4. Significant gene body polymerase changes IAA vs NT.
Supplementary Table S5. GSEA of PRO-Seq data from AIDW cells treated with IAA.

Supplementary Figures S1–S5 (appear on the following pages).

Supplementary Fig. S1- Page 1

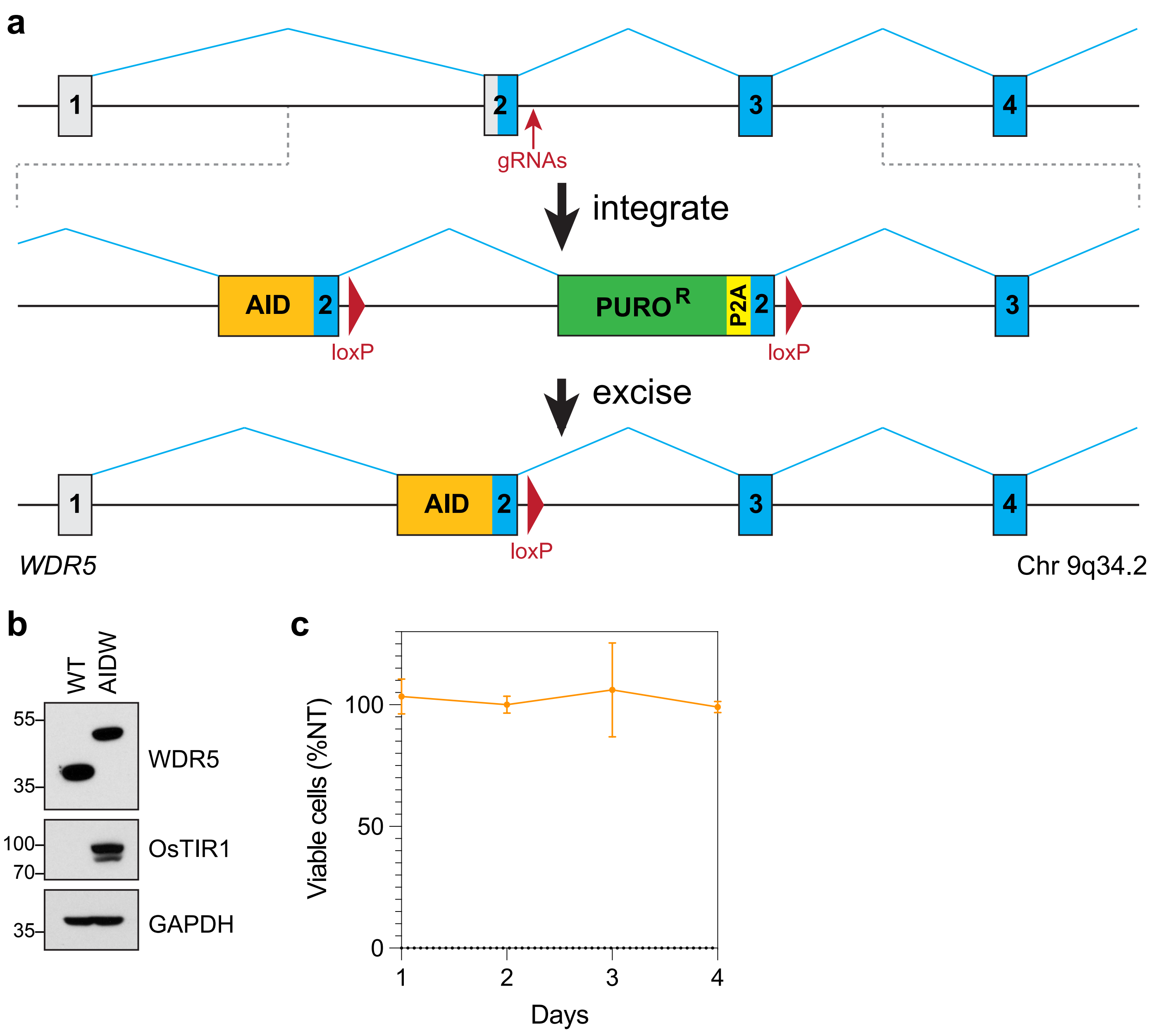

Supplementary Fig. S1- Page 2

d

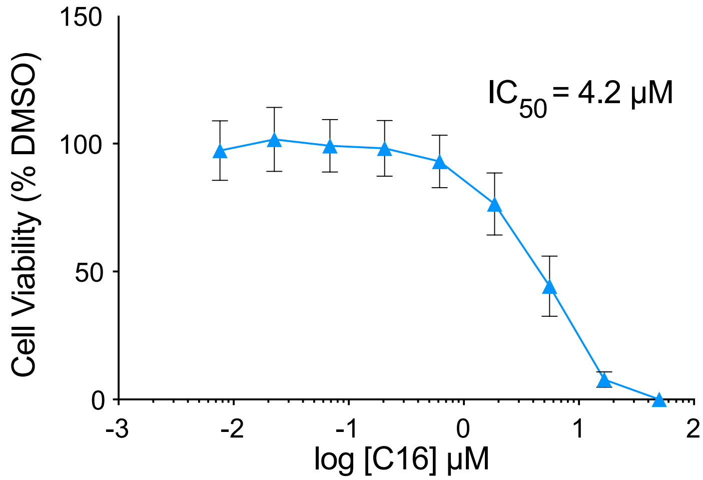

e

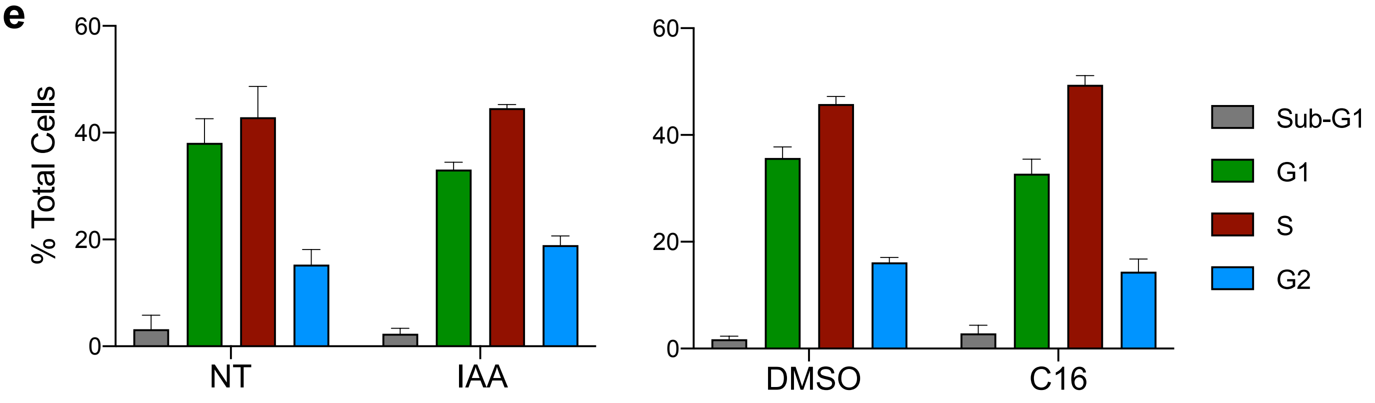

Supplementary Fig. S2 - Page 1

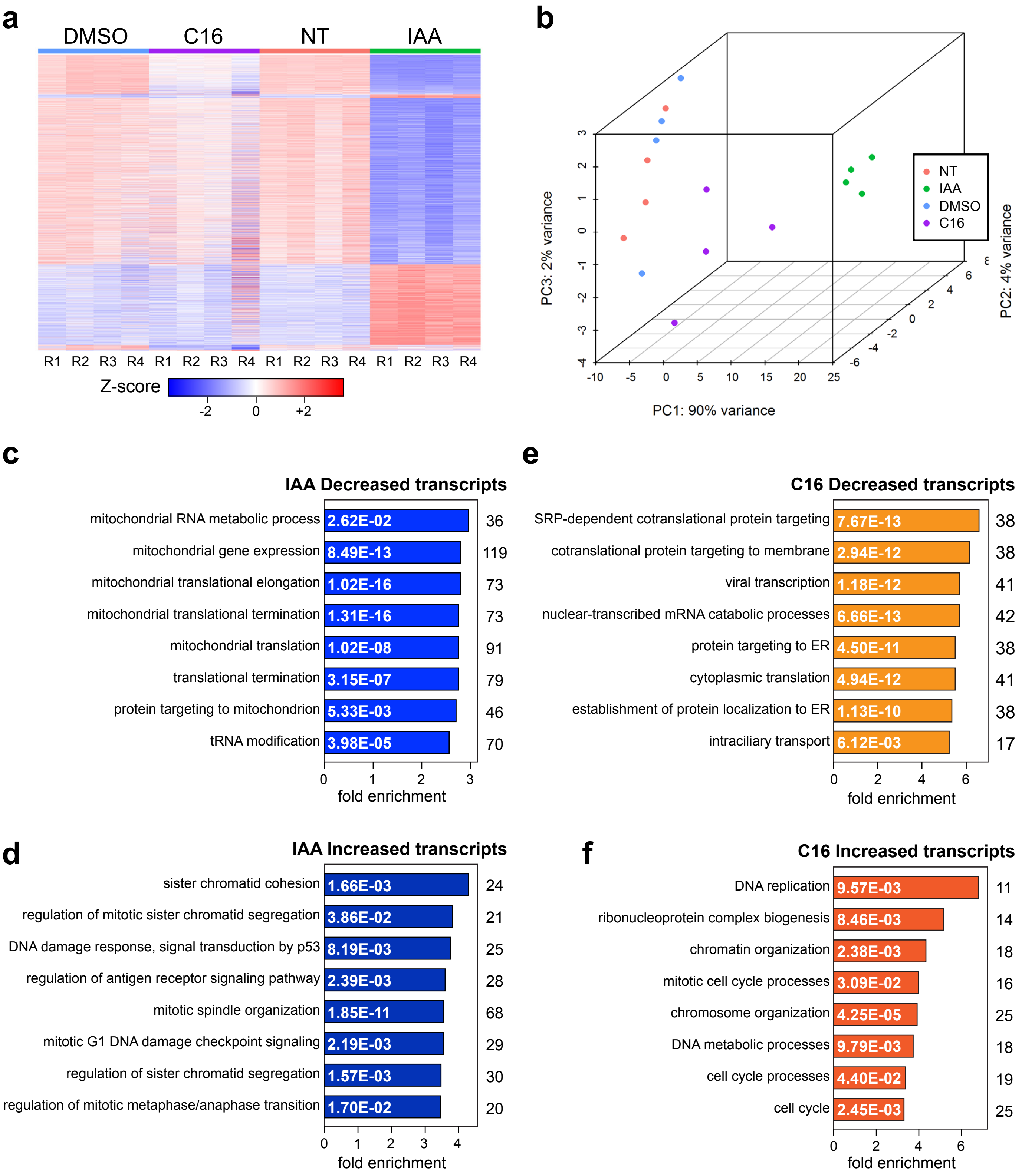

Supplementary Fig. S2 - Page 2

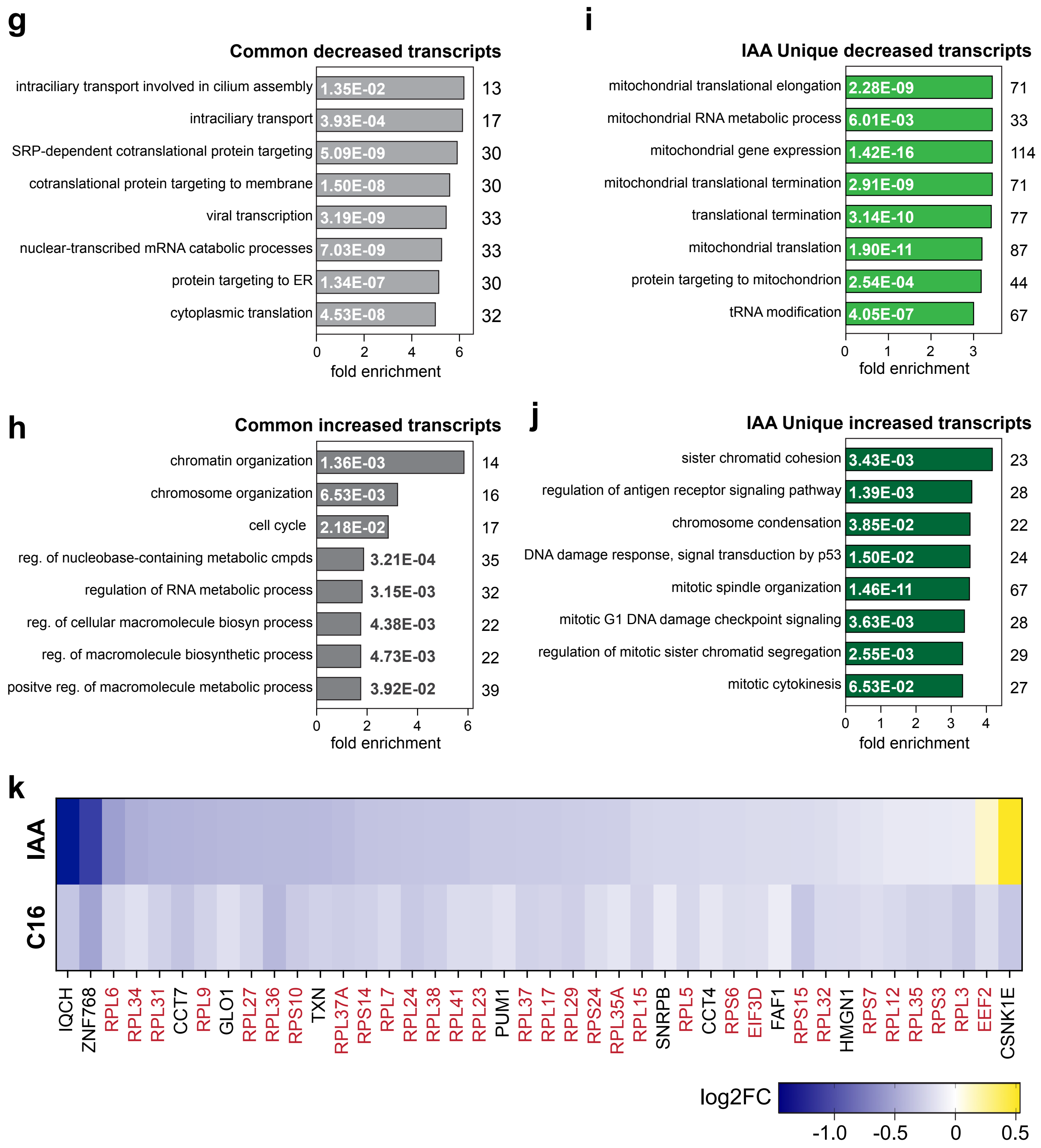

Supplementary Fig. S3 - Page 1

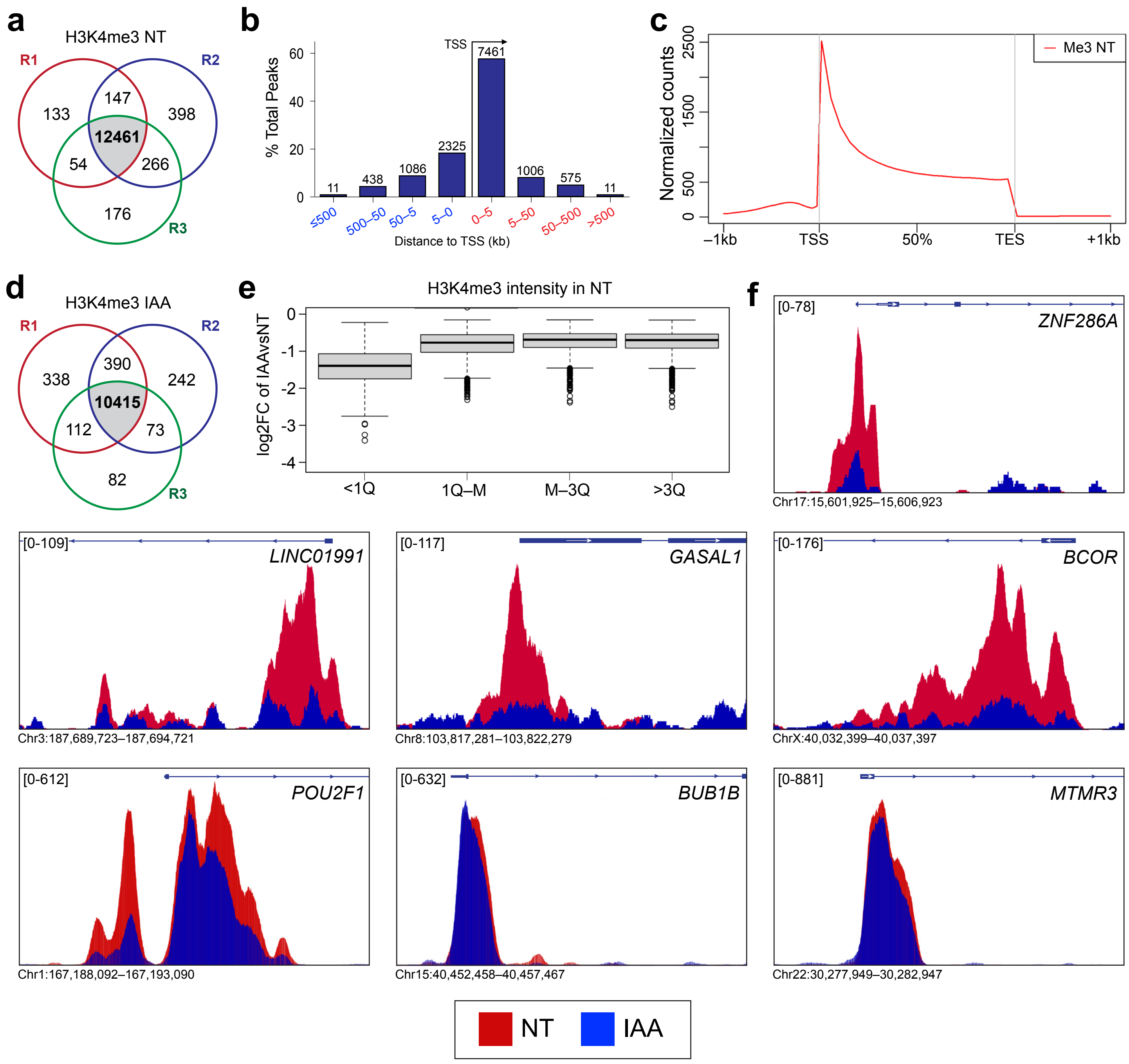

Supplementary Fig. S3 - Page 2

g

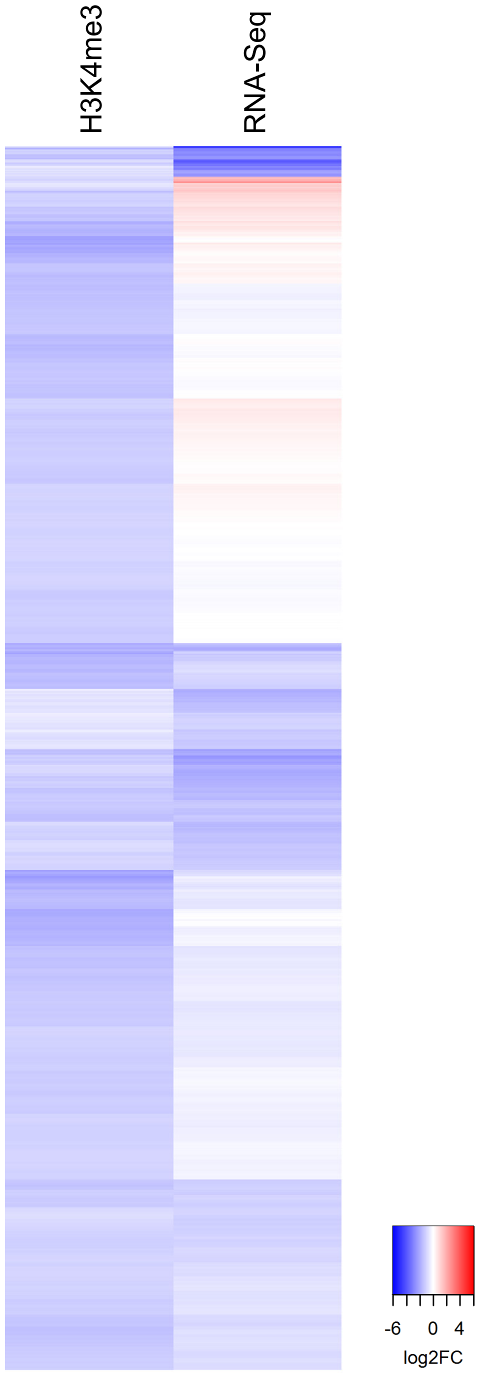

h

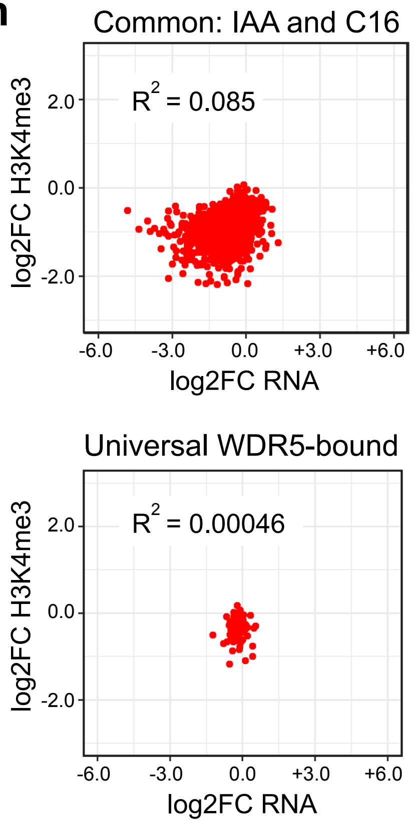

Supplementary Fig. S4 - Page 1

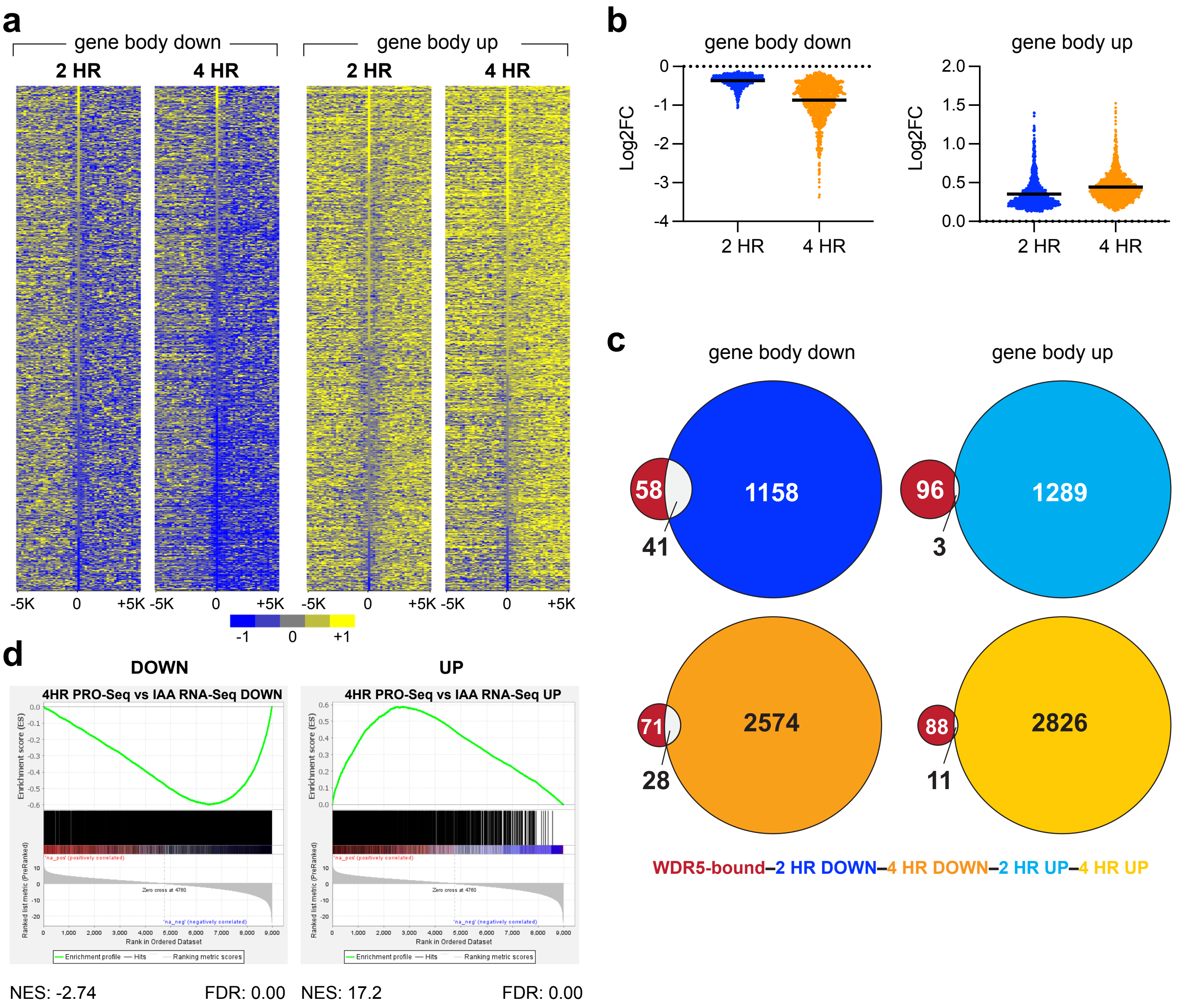

Supplementary Fig. S4 - Page 2

e

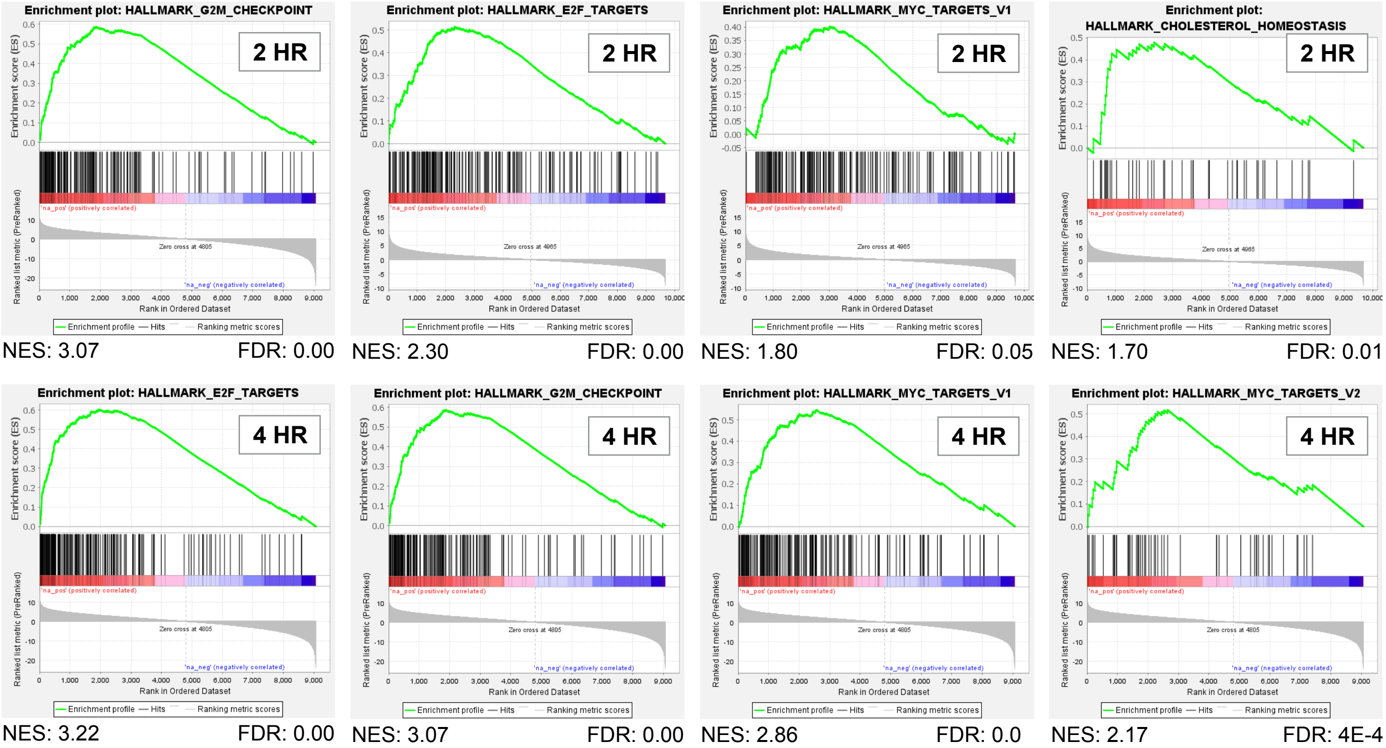

f

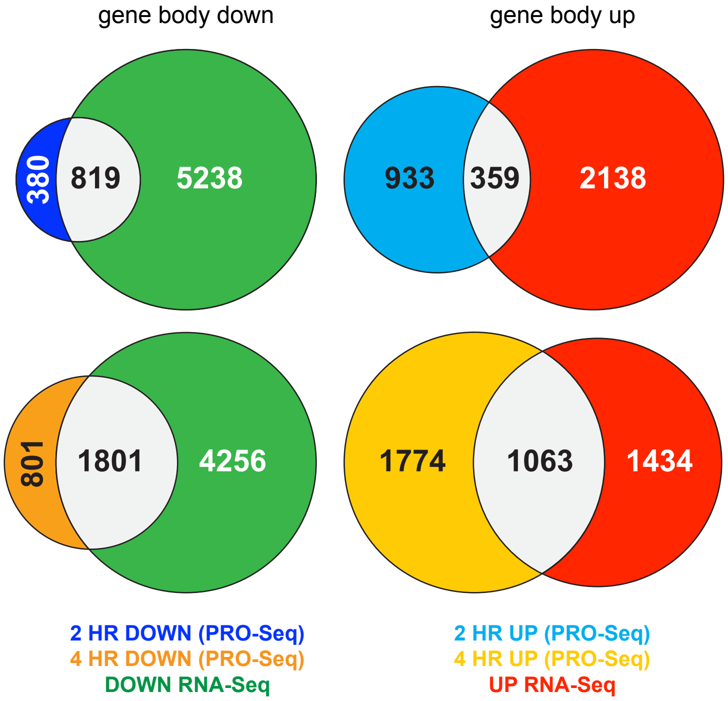

Supplementary Fig. S4 - Page 3

g

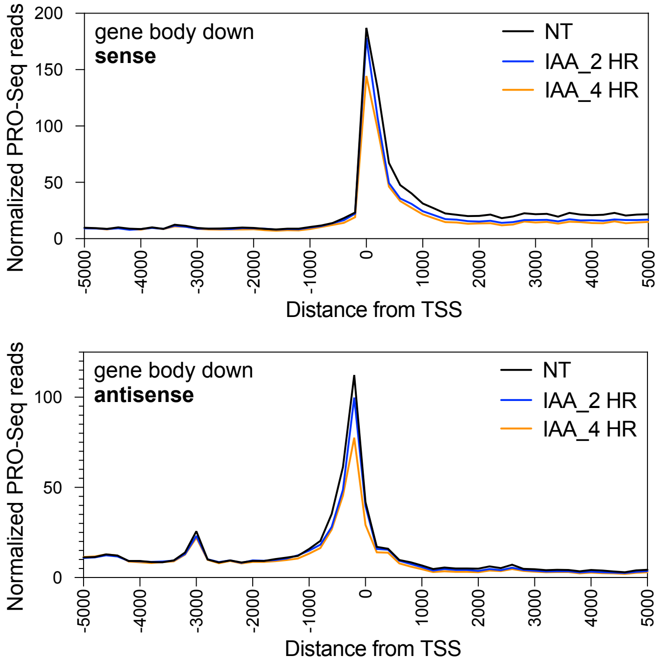

h

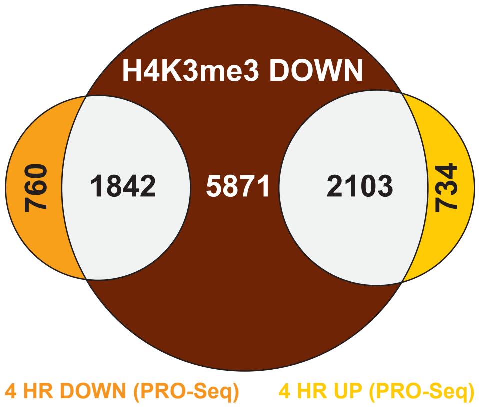

i

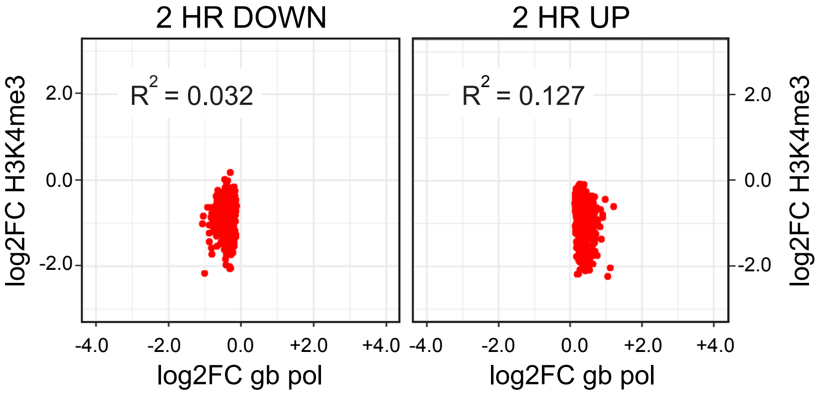

Supplementary Fig. S5- Page 1

Figure 1a

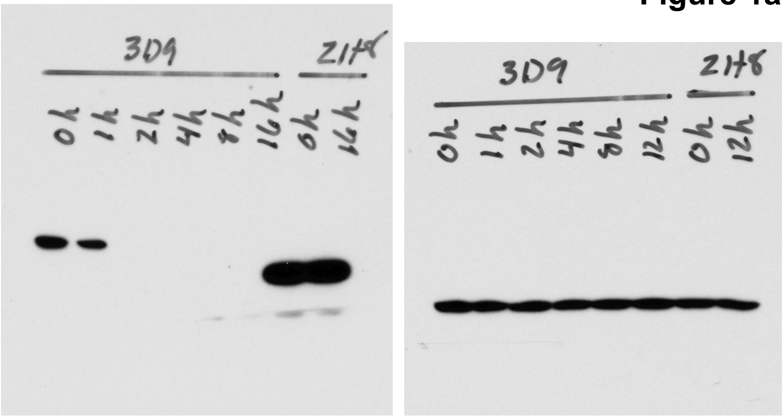

WDR5 GAPDH

Figure 1c

WDR5

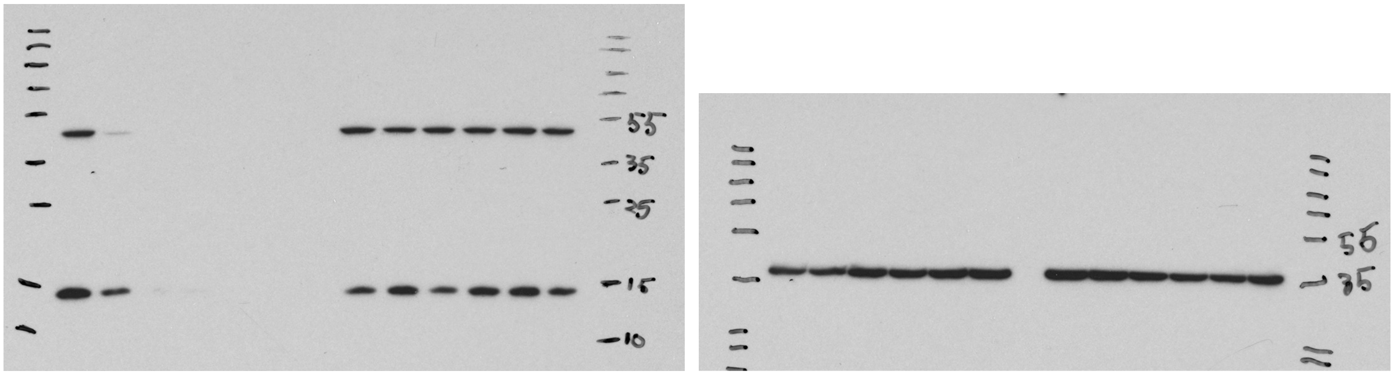

H3K4me3 GAPDH

p53 **Figure 1d—IAA treatment**

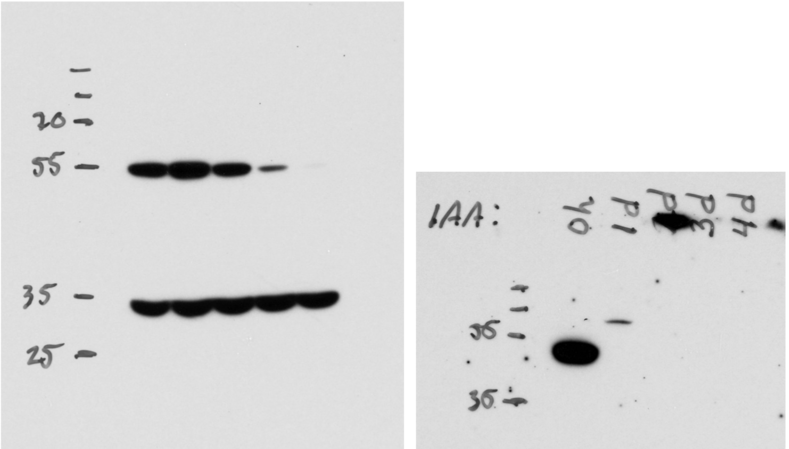

GAPDH WDR5

Supplementary Fig. S5- Page 2

Figure 1d—C16 treatment

p53

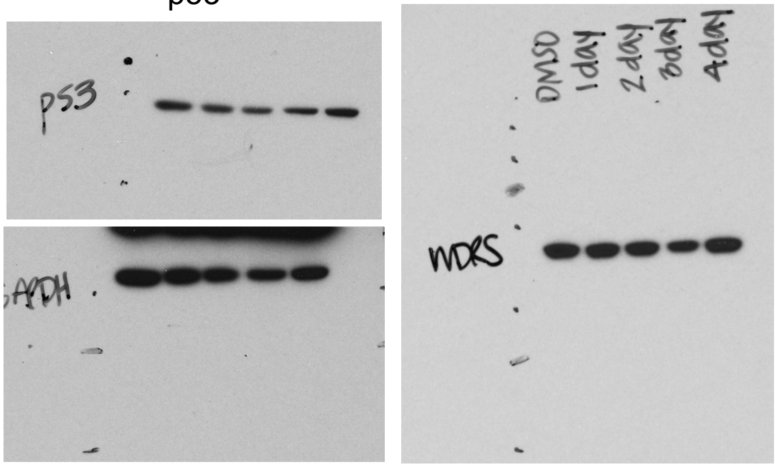

GAPDH

WDR5

Supplementary Figure S1b

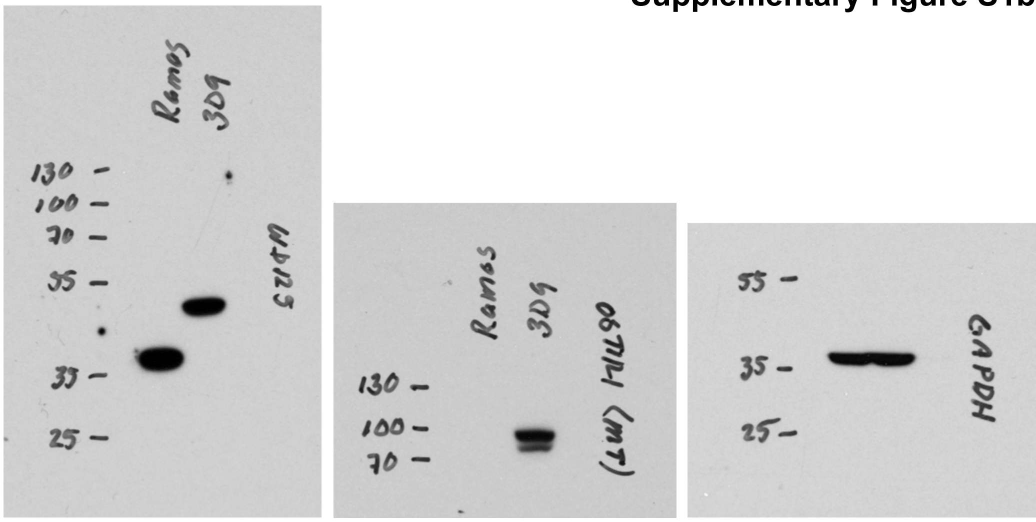

WDR5

OsTIR1

GAPDH
